# Quantitative proteomic analysis reveals AK2 as potential biomarker for late normal tissue radiotoxicity

**DOI:** 10.1186/s13014-019-1351-8

**Published:** 2019-08-09

**Authors:** Jérôme Lacombe, Muriel Brengues, Alain Mangé, Céline Bourgier, Sophie Gourgou, André Pèlegrin, Mahmut Ozsahin, Jérôme Solassol, David Azria

**Affiliations:** 1 IRCM, INSERM, University Montpellier, ICM, Montpellier, France; 2 ICM, Institut Cancer Montpellier, Montpellier, France; 3 CHU Vaudois, Lausanne, Switzerland; 4 Department of Pathology and Onco-Biology, CHU Montpellier, Montpellier, France; 5 Department of Radiation Oncology, ICM, 34298 Montpellier Cedex 5, France

**Keywords:** Radiotherapy, Normal tissue radiotoxicity, AK2, Radiation-induced breast fibrosis, Radiosensitivity, Proteomics, NADPH oxidases

## Abstract

**Background:**

Biomarkers for predicting late normal tissue toxicity to radiotherapy are necessary to personalize treatments and to optimize clinical benefit. Many radiogenomic studies have been published on this topic. Conversely, proteomics approaches are not much developed, despite their advantages.

**Methods:**

We used the isobaric tags for relative and absolute quantitation (iTRAQ) proteomic approach to analyze differences in protein expression levels in ex-vivo irradiated (8 Gy) T lymphocytes from patients with grade ≥ 2 radiation-induced breast fibrosis (grade ≥ 2 bf+) and patients with grade < 2 bf + after curative intent radiotherapy. Patients were selected from two prospective clinical trials (COHORT and PHRC 2005) and were used as discovery and confirmation cohorts.

**Results:**

Among the 1979 quantified proteins, 23 fulfilled our stringent biological criteria. Immunoblotting analysis of four of these candidate proteins (adenylate kinase 2, AK2; annexin A1; heat shock cognate 71 kDa protein; and isocitrate dehydrogenase 2) confirmed AK2 overexpression in 8 Gy-irradiated T lymphocytes from patients with grade ≥ 2 bf + compared with patients with grade < 2 bf+. As these candidate proteins are involved in oxidative stress regulation, we also evaluated radiation-induced reactive oxygen species (ROS) production in peripheral blood mononuclear cells from patients with grade ≥ 2 bf + and grade < 2 bf+. Total ROS level, and especially superoxide anion level, increased upon ex-vivo 8 Gy-irradiation in all patients. Analysis of NADPH oxidases (NOXs), a major source of superoxide ion in the cell, showed a significant increase of NOX4 mRNA and protein levels after irradiation in both patient groups. Conversely, only NOX4 mRNA level was significantly different between groups (grade ≥ 2 bf + and grade < 2 bf+).

**Conclusion:**

These findings identify AK2 as a potential radiosensitivity candidate biomarker. Overall, our proteomic approach highlights the important role of oxidative stress in late radiation-induced toxicity, and paves the way for additional studies on NOXs and superoxide ion metabolism.

**Electronic supplementary material:**

The online version of this article (10.1186/s13014-019-1351-8) contains supplementary material, which is available to authorized users.

## Background

Nowadays, radiation therapy (RT) is a major cancer treatment, and approximately 50–60% of patients undergo RT after primary cancer diagnosis [1]. Its success depends mainly on the total radiation dose homogeneously delivered within the target volume. However, RT use for cancer treatment inevitably involves exposure of the surrounding normal tissues, and may cause late and irreversible toxicities. Stratifying patients according to their risk level for such toxicities and modulating radiation dose in function of the sensitivity of the surrounding normal tissues would provide an invaluable tool for personalized RT and long-term patient management [2].

Many efforts have been made to develop assays to predict susceptibility to radiation injury with the ultimate objective of personalizing RT protocols [3, 4]. One of the most promising approaches for clinical use is the radiation-induced CD8 lymphocyte apoptosis (RILA) assay developed by our group several years ago [5, 6]. This assay is based on the measurement of the radiation-induced apoptosis rate in T lymphocytes isolated from a whole blood sample, 2 days after ex-vivo exposure to 8 Gy of radiation. Although RILA displays a low positive predictive value, its sensitivity and negative predictive value are high. Indeed, we and others consistently reported low RILA values in all patients with radiation-induced late toxicity, and high RILA values in all patients without late toxicities [7–10].

Many proteomics studies have shown proteome changes after irradiation; however, only few of them correlated these changes with radiation toxicity, especially in humans. Indeed, several models (in vitro, animals, humans, etc.) allowed the identification of radiation-induced alterations in the protein levels of tissues and bio-fluids, but only focused on dose- or time-related effects [11]. Other studies investigated protein expression changes in association with response to RT and tumor radioresistance in head and neck, breast, lung, and prostate cancer [12–19]. Overall, RT-induced proteome changes and their possible implication as predictors of radiation disease have been extensively reviewed [20–22]. However, due to the lack of clinical data and access to samples, few proteomic-based studies could identify human biomarkers predictive of radiation-induced damage in normal tissue. Only targeted proteomic approaches demonstrated the interest of plasma cytokines, particularly TGF-β1 [23–26], for the prediction of radiation-induced lung toxicity, although this is still a matter of debate [27, 28]. In the present study, we wanted to identify and characterize candidate proteins that might predict the risk of occurrence of grade ≥ 2 radiation-induced breast fibrosis after curative-intent RT. To this aim, we used the isobaric tags for relative and absolute quantitation (iTRAQ) proteomic approach and ex-vivo irradiated T lymphocytes (same protocol as for the RILA assay) from patients with grade ≥ 2 and with grade < 2 radiation-induced breast fibrosis. Overall, this approach revealed that oxidative stress may contribute to the mechanisms involved in the development of late radiation-induced toxicity.

## Methods

### Patient selection and toxicity assessment

Patients were selected from two prospective clinical trials (COHORT and PHRC 2005) and accepted to participate to this translational sub-study initially planned in both protocols. These trials were approved by our local ethics committee and registered at clinicaltrials.gov (NCT00208273 and NCT00893035, respectively). Skin toxicity was assessed according to the Common Toxicity Criteria Adverse Event version 3.0 (CTCAE v3.0) (11) at baseline, every week during radiotherapy, 3–6 weeks after the last radiotherapy fraction, every 3 months up to month 24, every 6 months during the first 3 years, and every year thereafter. Breast fibrosis was evaluated (as the primary endpoint) in 150 (phase II randomized COHORT study) and 502 (longitudinal PHRC 2005 trial) women with stage I-II breast cancer treated by adjuvant radiotherapy (7, 8). Consecutive patients who presented with late radiation-induced toxicity were selected and asked to participate to this study. In regards to the patients presenting no toxicity, the selection was made by performing a statistical match with the patients presenting toxicities, based on their clinical and treatment characteristic and RILA values. In both studies, patients underwent breast-conserving surgery with sentinel node biopsy and with or without axillary dissection. Before any adjuvant treatment, the RILA assay was performed for all patients. For this study, a total of 30 patients were selected within the follow-up period: two patients who developed grade ≥ 2 radiation-induced sub-cutaneous breast fibrosis (bf+) with low RILA score (≤16%) in the COHORT trial (discovery and confirmation sets); five patients with grade ≥ 2 bf + and low RILA score (≤16%) in the PHRC 2005 study (confirmation set); and 23 patients (COHORT and PHRC 2005) without grade ≥ 2 bf + and low (≤16%, *n* = 13) or high RILA score (> 16%; *n* = 10). Clinical information on these 30 patients is in Additional file 1: Table S1. Blood samples were collected in BD Vacutainer EDTA tubes. All samples were processed and stored in Montpellier (France).

### Proteomics analysis

#### Experimental design

**Fig. 1:**
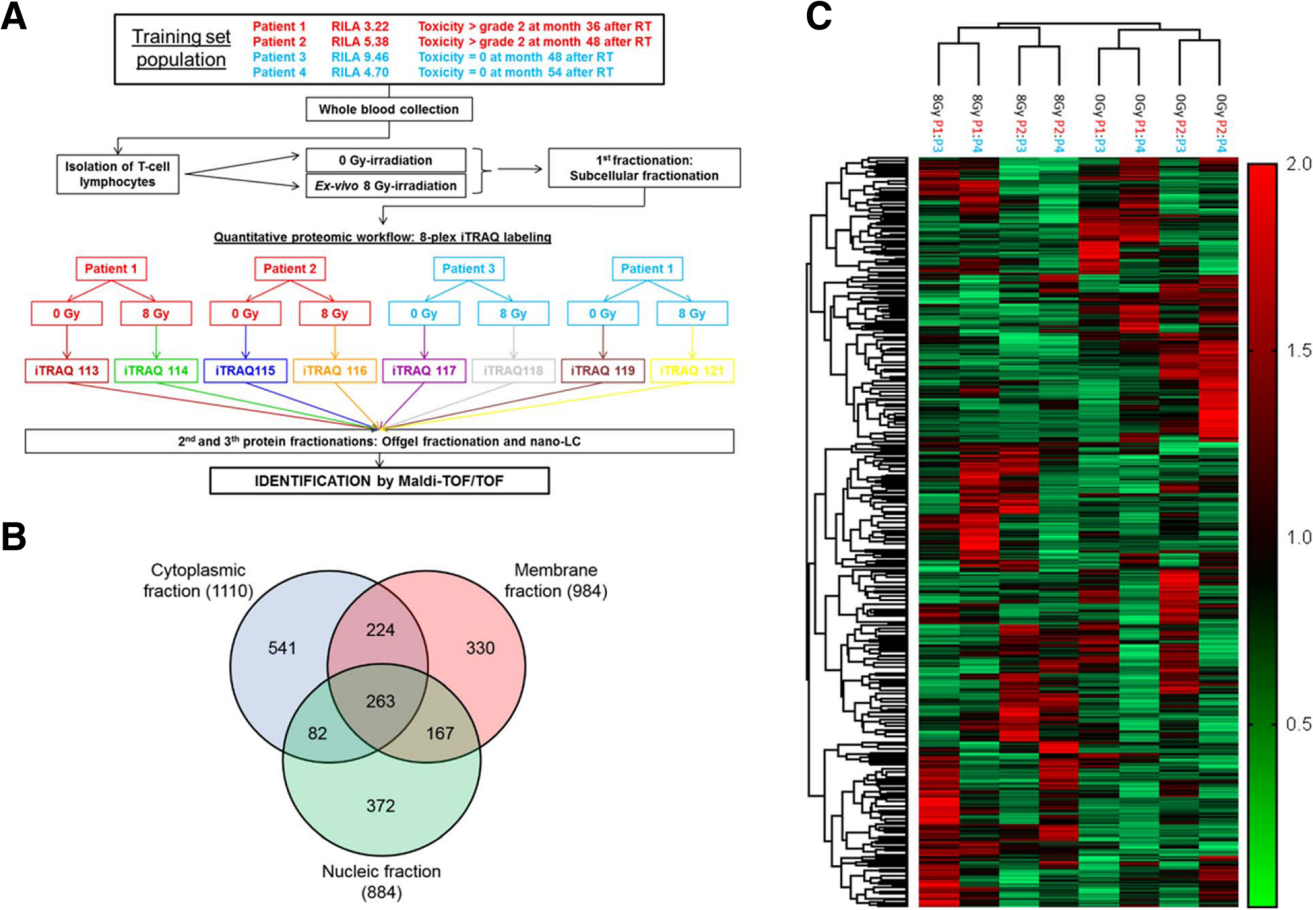
Quantitative Proteomic Analysis. **a** Schematic overview of the strategy used for the iTRAQ analysis. **b** Venn diagram showing the distribution of the 1979 identified proteins in each subcellular fraction. **c** Heat map and patient clustering according to the protein expression profiles. Each row represents one protein; columns represent the T lymphocyte samples of patients with (*n* = 2) and without (*n* = 2) grade ≥ 2 bf + after irradiation (8 Gy) or not (0 Gy). In **a**: eliminate population, change into T lymphocytes

The experimental design used for the iTRAQ-based quantitative proteomic analysis is illustrated in Fig. 1a. For the discovery stage, four patients with low RILA score (≤16%) were selected: two patients who developed grade ≥ 2 bf + 2 years after RT end, and two patients without any toxicity 4 years after RT end. Two technical replicates were performed for the mass spectrometry analysis. To identify new radiosensitivity biomarkers, first protein expression levels were compared in ex-vivo irradiated (8 Gy) T lymphocytes from these two groups of patients. Then, among the proteins that were differentially expressed (i.e., potential markers), the analysis focused on proteins the expression of which was modulated by ex-vivo irradiation (8 Gy) after comparison with non-irradiated (0 Gy) paired samples.

#### Sample preparation

T lymphocytes were purified immediately after collection by negative selection (RosetteSep, StemCell Technologies) from whole blood, and cultured in 6-well plates (4 × 10^6^ cells per well) in growth medium (RPMI medium containing 20% fetal bovine serum [FBS]) for 24 h. Then, half of each cell sample was irradiated ex-vivo (8 Gy), as done for the RILA assay (8). Forty-eight hours after irradiation, subcellular fractionation (cytosolic, membrane/organelle and nucleic fractions) was performed using the ProteoExtract Subcellular Proteome Extraction Kit (Calbiochem), according to the manufacturer’s recommendations and using the fraction-specific buffers provided in the kit. Protein concentration was measured with the Micro BCA Kit (Pierce).

#### iTRAQ labeling and peptide processing

iTRAQ labeling was performed according to the manufacturer’s protocol (AB Sciex). Briefly, 50 μg of proteins from each fraction was precipitated using four volumes of acetone. Pelleted proteins were dissolved in 500 mM triethylammonium bicarbonate/1% SDS. Proteins were reduced with 5 mM tris-(2-carboxyethyl) phosphine, alkylated with 10 mM methyl methanethiosulfonate, and digested with trypsin overnight. The resulting peptides were labeled by incubation with one of the eight isobaric amine-reactive tags for 2 h. Labeled peptides were combined and cleaned using a strong cation exchange cartridge, as recommended by the manufacturer (AB Sciex). The strong cation exchange eluents were desalted using an Oasis HLB extraction cartridge (Waters Corporation) and vacuum-dried before isoelectric focusing separation of peptides using the Agilent 3100 OFFGEL Fractionator (Agilent). Briefly, one IPG DryStrip (24 cm, pH 3–10) was rehydrated with 20 ml/well of a solution containing 0.25% IPG buffer, pH 3–10 (GE Healthcare), for 15 min. Desalted peptides were dissolved in 0.25% IPG buffer, pH 3–10, and the peptide solution (150 ml) was pipetted into each well (24 wells). Isoelectric focusing separation was performed at 50 kVh, with a maximum current of 50 mA and power of 200 mW. Fractions were purified using OMIX C18 100 ml pipette tips (Varian). Then, peptides were eluted and lyophilized before reconstitution for the 2D nano-LC/MS/MS analysis.

#### Mass spectrometry analysis

Peptides were separated using an Acclaim PepMapTM (C18, 3 μm, 100 Å), 75 μm/15 cm column (Dionex—LC Packing) and the Ultimate 3000 nano-LC system coupled to a ProbotTM Microfraction Collector (Dionex). The used mobile phases were: 2% ACN with 0.05% TFA (A), and 80% ACN with 0.05% TFA (B). The gradient elution steps were performed with a flow rate of 0.3 ml/min as follows: 0–50% B for 60 min, 50–80% B for 30 min, 80–100% B for 5 min, and then 100% B for 10 min. Fractions were mixed directly with the MALDI matrix solution (2 mg/ml acyano-4-hydroxycinnamic acid in 70% ACN with 0.1% TFA) at a flow rate of 1.2 ml/min. Fractions were spotted onto the Opti-TOFTM LC/MALDI insert plates (AB Sciex) using the Probot spotting device during 110 min at a speed of 11 s per well. Plates were analyzed using a MALDI TOF/TOF 4800 mass spectrometer (AB Sciex). MS spectra from m/z 700 to 4000 were acquired in positive reflector ion mode using 1500 laser shots. The 10 most abundant peptide precursor ions with signal-to-noise ratios higher or equal to 50 were selected for MS/MS analysis using 3500 laser shots from m/z 300–1500.

#### Data analysis

Protein identification and quantification were performed with the ProteinPilotTM software 2.0.1 and the Paragon method (AB Sciex). The obtained MS/MS spectra were searched against the UniProtKB/Swiss-Prot database (release version 2010_08, http://www.uniprot.org). The search parameters for tryptic cleavage and accuracy were built-in functions of the software. The other data analysis parameters were as follows: 8-plex iTRAQ peptide labeling, cys-alkylation by MMTS, biological modifications, and a thorough identification search. The local false discovery rate (FDR) was estimated using the Proteomics System Performance Evaluation Pipeline (PSPEP) tool. Proteins containing one or more peptides with high confidence score (> 95%) and low FDR (estimated local FDR of 5%) were considered positively identified. iTRAQ labeling followed by nano-LC MS/MS analysis was repeated in duplicate to reduce the effect of experimental variation.

#### Statistical analysis and gene ontology analysis

The following criteria were required to select a protein for further analysis: two or more unique peptides with a high confidence score (> 99%), a *p*-value for protein quantification assigned by the ProteinPilot software < 0.05, and > 1.5-fold difference relative to the control sample. For this discovery phase, the p-value was not corrected with a more stringent statistical analysis, such as adjustment for multiple testing, and the risk for false discovery association still existed at this step. The gene ontology (GO) classification was determined using the DAVID bioinformatics resources (12). Statistical significance was determined using GraphPad Prism version 7.00 for Windows, GraphPad Software, La Jolla California USA, www.graphpad.com.

#### Immunoblotting

For the confirmation by western blot analysis, blood samples from five patients with grade < 2 bf + and five patients with grade ≥ 2 bf + were included. Because of the limited number of patients with grade ≥ 2 bf+, the two patients used in the discovery phase were also included in this analysis. Whole blood samples were treated as described for the quantitative proteomic analysis except for protein extraction that was performed using the radioimmunoprecipitation assay (RIPA) buffer. Proteins were eluted by adding 100 μl 2× SDS-PAGE buffer, and heated at 95 °C for 10 min. Samples were size-separated by electrophoresis on SDS-polyacrylamide gels (12%), and transferred to PVDF membranes (Invitrogen). Membranes were blocked with PBST (PBS plus 0.1% Tween-20) containing 5% non-fat milk at 25 °C for 60 min. Blots were then incubated (4 °C, overnight) with primary antibodies against adenylate kinase 2 (AK2) (1/100, sc-28,786; Santa Cruz Biotechnology), annexin A1 (ANXA1) (1/100, sc-11,387; Santa Cruz Biotechnology), galectin-1 (LSGAL1) (1/100, sc-19,277; Santa Cruz Biotechnology), heat shock cognate 71 kDa protein (HSPA8) (1/500, sc-7298; Santa Cruz Biotechnology) and isocitrate dehydrogenase 2 (IDH2) (1/200, sc-134,923; Santa Cruz Biotechnology). After five washes (5 min/each) with PBST, blots were incubated with an anti-rabbit HRP-conjugated antibody (1:2500, Invitrogen), or an anti-mouse HRP-conjugated antibody (1:10000, Jackson ImmunoResearch) at 25 °C for 1 h. After washing five times at 25 °C with PBST (5 min/each), blots were developed with ECL Plus (Amersham). β–actin was used as loading control (1/500, sc-47,778, Santa Cruz Biotechnology). Differences between experimental conditions for immunoblotting results were analyzed using the Mann-Whitney test. *P* values < 0.05 were considered statistically significant.

### Functional analysis

#### Peripheral blood mononuclear cell (PBMC) isolation

Whole blood samples were collected in EDTA Vacutainer tubes from 20 patients: 7 with grade ≥ 2 bf + (including the 5 patients from the proteomics analysis/immunoblotting) and 13 patients with grade < 2 bf+. PBMCs were immediately purified by density gradient centrifugation (Ficoll-Paque PLUS, GE Healthcare Life Sciences) according to the manufacturer’s recommendations.

#### ROS and superoxide anion quantification

PBMCs were resuspended in RPMI-1640 medium (Sigma) with 20% FBS, and seeded in 6-well plates at a concentration of 4 × 10^6^ cells per well. The day after, half of the plates were irradiated (8 Gy). At 6, 24, 48, 72 h and 120 h after irradiation, the production of ROS–RNS and superoxide anion in irradiated and control (0 Gy) cells was measured using the Total ROS/Superoxide Detection Kit (Enzo Life Sciences AG) with a slight modification of the fluorescence microplate assay protocol for cells in suspension. Briefly, 1 × 10^6^ cells were washed once, and incubated in 1 ml of buffer containing 2 μM oxidative stress detection reagent (green) and 2 μM superoxide detection reagent (orange) for 60 min. Then, 100 μl of cell-containing mixture was seeded in 96-well black plates (7 aliquots for each sample), and fluorescence was quantified as described in the original protocol. Cell number and viability were assessed with the trypan-blue dye-exclusion assay.

#### Total RNA extraction and quantitative RT-PCR analysis

Total RNA was purified from PBMCs 24 h after irradiation using the RNAeasy Mini Kit (Qiagen) according to the manufacturer’s instructions. RNA quantity was assessed with a NanoDrop 2000 Spectrophotometer (ThermoScientific). For first-strand cDNA synthesis, 500 ng of RNA in 12 μl of RNase-free water was denaturated at 65 °C for 10 min. Then, 2 μl of 10× first-strand buffer, 2 μl of 5 mM dNTPs, 0.4 μl oligodT (50 μM), 40 UI of RNaseOUT Recombinant Ribonuclease Inhibitor (Invitrogen, Carlsbad, CA, USA), and 200 Units of Superscript III (Invitrogen) were added to a total volume of 20 μl. The mixture was incubated at 37 °C for 60 min, and then at 95 °C for 5 min. Complementary DNA was frozen at − 20 °C until use. Real-time quantitative PCR (qRT-PCR) was performed with the LightCycler 480 SYBR Green I Master system (Roche Applied Science) in a final volume of 10 μl, including 0.5 μl of each primer (0.25 μM), 5 μl of the supplied enzyme mix, 3 μl of H_2_O, and 1 μl of template (1:20 dilution). After pre-incubation at 95 °C, 45 cycles were run as follows: 95 °C for 15 s, 60 °C for 20s, and 72 °C for 10s. All samples were run in triplicate in 384-well optical PCR plates (Roche Diagnostics). Each PCR run included a no-template control (water added instead of cDNA). For each gene, all samples were tested in the same plate. The melting curves of the PCR products were analyzed using the LightCycler software to exclude amplification of non-specific products. Results were normalized to the RS9 and β2-microglobulin housekeeping gene transcripts (Additional file 2: Table S2). Differences in NADPH oxidase (NOX) mRNA levels between control (0 Gy) and irradiated (8 Gy) samples were analyzed with the Wilcoxon matched-pairs signed rank test, whereas differences between patient groups were analyzed with the two-way analysis of variance (ANOVA) with the Bonferroni post hoc test. For all figures in which error bars are shown, data represent the mean ± SEM. Statistical outliers and specimens with measurement errors were excluded.

#### Immunoblotting

Protein extraction from PBMCs and western blot analysis were performed following the same protocol used for the proteomics analysis confirmation. PVDF membranes were incubated with an anti-NOX4 antibody (1/200, sc-30,141; Santa Cruz Biotechnology). NOX4 level was normalized to β-actin level and differences between groups were analyzed using the paired t-test.

## Results

Identification and quantification of differentially expressed proteins in patients with and without grade ≥ 2 breast fibrosis (bf+).

To identify proteins that might predict the risk of grade ≥ 2 bf+, we compared the proteomic data of control (0 Gy) and irradiated (8 Gy) T lymphocytes from patients with (*n* = 2) and without (*n* = 2) grade ≥ 2 bf + obtained by 2D nano-LC/MS/MS analysis after iTRAQ labeling. The proteomic workflow is shown in Fig. 1a. We also performed subcellular fractionation to maximize the likelihood of identifying proteins. Using stringent criteria, including one or more peptides with a > 95% confidence score and 5% local FDR, we identified 1110, 984 and 884 proteins in the cytosolic, membrane and nucleic fraction, respectively (Fig. 1b), of which 263 were shared by the three subcellular fractions. In total, we identified and quantified 1979 non-redundant proteins for comparison between patients with or without grade ≥ 2 bf + (Fig. 1c). We classified (subcellular distribution and biological processes) these 1979 proteins using the GO classification system. Specifically, 33% of the identified proteins were cytoplasmic proteins, 27% were membrane proteins, 23% were nuclear proteins, 11% were mitochondrial proteins, and 6% were cytoskeletal proteins (Additional file 3: Figure S1A). We also assessed the subcellular distribution of the different fractions to confirm the specific protein enrichment for each fraction (Additional file 4: Figure S2). Proteins were associated with a broad range of biological processes (Additional file 3: Figure S1B): 23% of proteins were involved in regulation of biological processes, 19% in response to stimulus, 13% in metabolic processes, and 12% in cell communication. The other proteins were associated with various cellular functions, including immune system response, transcription, transport, cell death, and oxidation-reduction processes. We then compared the expression profile of these 1979 unique proteins in each control (0 Gy) and irradiated (8 Gy) sample pair from patients with and without grade ≥ 2 bf + to identify differentially expressed proteins related to late radiation toxicity. First, we selected proteins using stringent biological selection criteria: i) proteins identified with at least two peptides with high confidence (99%); and ii) proteins significantly (*p*-value < 0.05) and differentially (fold-change ≥1.5 or ≤ 0.66) expressed in irradiated T lymphocytes of the two patients with grade ≥ 2 bf + relative to the irradiated samples from the two patients without grade ≥ 2 bf+. These criteria were fulfilled by 23 proteins, mainly involved in oxidation-reduction processes and RNA processing (Additional file 5: Figure S3). Then, among these 23 proteins, we selected 5 proteins with high 8 Gy/0 Gy ratio in the two patients with grade ≥ 2 bf + relative to the two patients without grade ≥ 2 bf+: adenylate kinase 2 (AK2), annexin A1 (ANXA1), galectin-1 (LSGAL1), heat shock cognate 71 kDa protein (HSPA8), and isocitrate dehydrogenase 2 (IDH2) (Additional file 6: Table S3).

### Confirmation of the iTRAQ data by western blotting and qRT-PCR analysis

**Fig. 2:**
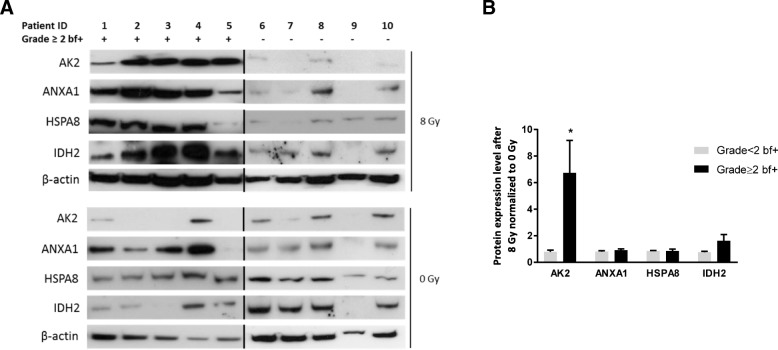
AK2 is differentially expressed between patients with and without grade ≥ 2 bf+. **a** Immunoblot analysis of AK2, ANX1, HSPA8 and IDH2 in control (0 Gy) and 8 Gy-irradiated T lymphocytes from the indicated patients. β-actin was used as loading control. **b** Quantification of the immunoblot presented in A using the ImageJ software. Data are the mean ± SEM; *p < 0.05 (2-tailed Mann-Whitney test). qRT-PCR analysis of AK2 and IDH2 mRNA expression in control (0 Gy) and 8 Gy-irradiated T lymphocytes from the same patients with and without grade ≥ 2 bf+. The graph shows the expression level in irradiated samples relative to non-irradiated samples

To confirm the proteomic results, we assessed (western blotting) AK2, ANXA1, HSPA8, IDH2 and LSGAL1 expression in control (0 Gy) and irradiated (8 Gy) T lymphocytes from five patients with grade ≥ 2 bf + and five patients with grade < 2 bf + (Fig. 2a). Unfortunately, we could not quantify LSGAL1 expression due to antibody malfunction. This analysis showed that only AK2 (*p* = 0.0419) was significantly overexpressed in irradiated samples from both patient groups (Fig. 2b). Conversely, AK2 mRNA level was comparable between groups, supporting the hypothesis of a role of protein stability rather than a transcriptomic effect in late toxicity (Additional file 7: Figure S4).

### Free radical level and NADPH oxidase quantification in PBMCs

Our proteomic analysis revealed that many proteins involved in oxidative stress regulation were differentially expressed between patients with and without grade ≥ 2 bf+. Oxidative stress and especially free radicals are induced by ionizing radiation and contribute to radiation injury [29]. Therefore, we quantified the total intracellular level of ROS and superoxide ion using a specific cell-permeable fluorescent probe. Due to the limited amount of blood samples and the need of an important number of cells for this experiment, we isolated PBMCs instead of T lymphocytes from 20 patients (*n* = 7 with grade ≥ 2 bf + and *n* = 13 with grade < 2 bf+). We measured ROS and superoxide ion levels at different time points (from 6 to 120 h) after irradiation. As expected, total ROS level and especially superoxide anion increased progressively after ex-vivo irradiation (Additional file 8: Figure  S5) in all patients. However, total ROS and superoxide anion levels were not different between patients with grade ≥ 2 bf + and grade < 2 bf+, with the exception of a slight increase of total ROS level at 72 h after irradiation in patients with grade ≥ 2 bf + (Additional file 9: Figure S6).

**Fig. 3:**
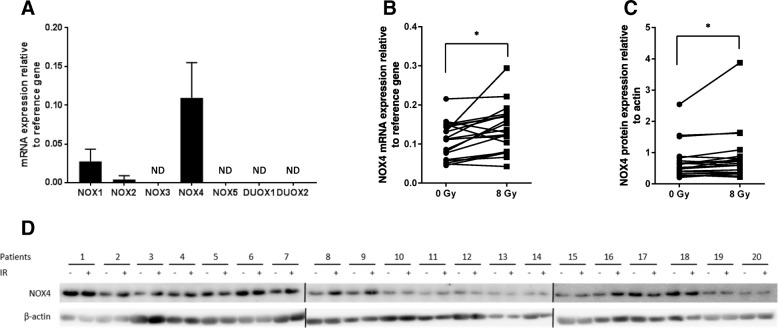
NOX4 mRNA and protein levels are increased in PBMCs after ex-vivo irradiation. **a** mRNA expression of NOX family members in all patients (*n* = 20; *n* = 7 with and *n* = 13 without grade ≥ 2 bf+) in non-irradiated PBMC samples was analyzed by qRT-PCR. ND, not detected (below threshold). **b** NOX4 mRNA expression is increased in all patients (*n* = 20) at 24 h after irradiation (8 Gy) compared with non-irradiated samples (0 Gy) (Wilcoxon matched-pairs signed rank test). **c** NOX4 protein expression in all patients (*n* = 20) at 24 h after irradiation (8 Gy) compared with non-irradiated samples (0 Gy) (Wilcoxon matched-pairs signed rank test). **d** Western blot analysis of NOX4 expression in all 20 patients before (−) and at 24 h after 8 Gy-irradiation (+). β-actin was used as loading control. Data are the mean ± SEM; *p < 0.05 (paired t-test)

**Fig. 4:**
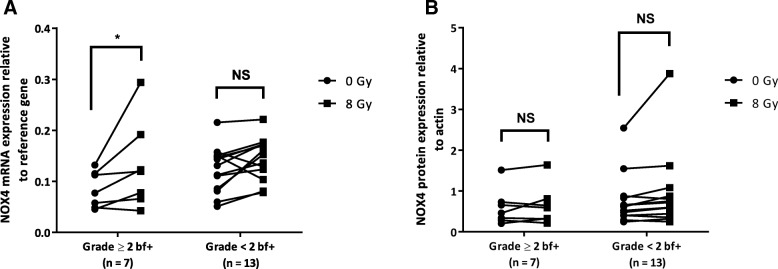
After irradiation (8 Gy), NOX4 mRNA level is significantly increased in PBMCs of patients with grade ≥ 2 bf+. **a** NOX4 mRNA expression (qRT-PCR analysis) in patients with grade < 2 bf + (*n* = 13) and with grade ≥ 2 bf + (*n* = 7) before (circles) and at 24 h after 8 Gy-irradiation (squares). **b** NOX4 protein expression (western blotting) in patients with grade < 2 bf + (*n* = 13) and grade ≥ 2 bf + (*n* = 7) before (circles) and at 24 h after 8 Gy-irradiation (squares) (two-way ANOVA with Bonferroni post-tests). NS: Not Significant; *p < 0.05, by paired t-test. Data are the mean ± SEM

In cells, superoxide anion production is mainly catalyzed by NOXs through dioxygen reduction [30]. Therefore, we investigated the potential role of the superoxide-generating NOX family by measuring NOXs mRNA levels in PBMCs. Among all the tested isoforms, NOX4 was the most abundantly expressed in control (0 Gy) (Fig. 3a) and irradiated (8 Gy) PBMC samples (Additional file 10: Figure S7). Comparison of NOX4 mRNAs expression in control (0 Gy) and irradiated (8 Gy) PBMCs from all patients together showed a significant increase after irradiation (*p* = 0.0027) (Fig. 3b). NOX4 protein level also was increased after irradiation (*p* = 0.0153) (Fig. 3c-d). However, only NOX4 mRNA (but not protein) increase after irradiation was significantly higher in patients with grade ≥ 2 bf + than in patients with grade < 2 bf + (*p* = 0.022, Fig. 4a-b).

## Discussion

Although tumor radioresistance has been widely investigated, few studies have considered normal tissue radiosensitivity, which is a key factor in RT success [31]. Despite their numerous benefits, proteomic approaches for studying late radiation-induced toxicity have not been widely developed [31]. Some studies in animal models investigated proteome changes that may predict late radiotoxicity in heart [32, 33], brain [34], hepatic [35], and skin [36, 37]. However, to our knowledge, only few studies measured the basal level of plasma proteins in patients with non-small cell lung (NSCLC) before starting RT [38, 39]. The authors showed that the level of C4b-binding protein alpha chain, complement C3, and vitronectin was higher in patients who later developed grade ≥ 2 radiation-induced lung toxicity than in patients who did not. By using a bioinformatics approach, Oh et al. analyzed proteomic data obtained using plasma from 26 patients with locally advanced NSCLC, and identified α-2-macroglobulin protein as predictive biomarker for radiation-induced pneumonitis [40]. Despite the depletion of the most abundant plasma proteins, these studies still identified common differentially expressed proteins. However, we think that rather than assessing the protein basal level, the approach of looking at radiation-induced changes before starting RT is more promising, as successfully demonstrated by the RILA assay. Accordingly, in the present study, we used iTRAQ quantitative proteomics to identify lymphocyte proteins that might predict late radiation-induced toxicity. To this aim, we first selected proteins that were differentially expressed between patients with grade ≥ 2 bf + and grade < 2 bf+. Then, to ensure that the protein expression differences between groups were induced by radiation, we selected the proteins for which the four ratios between patients with grade ≥ 2 bf + and grade < 2 bf + after 8 Gy where higher than the four ratios for the non-irradiated patients’ samples. Using this approach, we identified 23 proteins, and confirmed that among these proteins, AK2 was differentially expressed in irradiated T lymphocytes from these two groups of patients. The main function of the mitochondrial protein AK2 is to monitor the cellular energy state through nucleotide signaling [41]. Some studies demonstrated that disturbed adenine nucleotide metabolism may lead to abnormal ROS production, and AK2 knockdown or AK2 mutation results in increased ROS levels [42, 43]. Besides AK2, our proteomic analysis showed that many proteins involved in oxidative stress homeostasis were differentially expressed between patients with and without grade ≥ 2 radiotoxicity. Oxidative stress is a key biochemical event during radiation exposure, and is often seen as the main mediator of the deleterious effects of ionizing radiation in cells. Oxidative changes may continue for days or months after the initial exposure to radiation, and the persistence of such chronic stress could explain most of the long-term side effects observed after RT [44]. Therefore, we measured the level of total ROS and of superoxide anion, the precursor of most ROS and a mediator in oxidative chain reactions [45]. Total ROS and particularly superoxide anion were increased in irradiated PBMCs from all patients. NOXs, which are a major source of superoxide anion in the cell [46, 47], form a family that includes seven enzymatic complexes (NOX1 to NOX5, DUOX1, and DUOX2). All NOX family members transport electrons across biological membranes to reduce oxygen to superoxide anion. Recent evidences indicate that some NOX isoforms are often responsible for ROS-induced fibroblast activation, and are involved in fibrosis development, the main form of radiation-induced late toxicity [48, 49]. To our knowledge, this is the first study that compared NOX expression in normal tissue between patients with and without grade ≥ 2 bf+. We focused on NOX4 because it was the most abundant isoform in PBMCs. We found that overall, both NOX4 mRNA and protein levels were increased after ex-vivo irradiation, suggesting a radiation-induced activation in mononuclear blood cells. Moreover, NOX4 mRNA level increase (compared with non-irradiated samples) was significant only in samples from patients with grade ≥ 2 bf+. However, NOX4 mRNA upregulation in patients with grade ≥ 2 bf + was not correlated with ROS level, and we did not identify any difference in superoxide anion and total ROS levels between patients with and without grade ≥ 2 bf+. This could be linked to the fact that the identity of the reactive oxygen product of NOX4 is currently a matter of debate. Differently from NOX1-NOX3 and NOX5 that release superoxide anion, some studies reported that H_2_O_2_ is the major product of NOX4, whereas other studies detected also superoxide anion [50]. Moreover, in samples from patients with grade ≥ 2 bf+, only NOX4 mRNA and not protein expression was increased, suggesting that irradiation affects only NOX4 transcription and not its protein activity. This could explain the absence of effect on ROS production. The biology of NADPH oxidases is complex and additional studies are needed to determine its role in late radiotoxicity. Finally, due to the difficulty to obtain samples from patients with late radiotoxicity, the limited number of analyzed samples does not allow clear conclusions about NOX4 role in individual radiosensitivity. An independent and prospective validation study with a larger number of patients is required.

To our knowledge, AK2 has never been directly involved in NOXs regulation. However, among the 23 candidate proteins, some contribute to NOX regulation, such as HSPA8 and APEX1. Indeed, proteins of the HSP70 family decrease ROS production by the NOX isoforms 1, 3, and 5, and bind to NOX2 [51]. APE1 directly controls the intracellular level of ROS through its inhibitory effect on Rac1, the NOX regulatory subunit [52]. In our study, we used T cells for the proteomic analysis that identified AK2, and PBMCs to measure NOX4 level after irradiation. Therefore, our data cannot be used to suggest a direct interaction.

Finally, as radiation-induced apoptosis may predict late toxicity, it is interesting to note that inhibition of AK2 and NOX4 reduces apoptosis [53–58]. Specifically, AK2 mediates mitochondrial apoptosis through the formation of an AK2–FADD–caspase 10 complex [53, 54], whereas NOX4-mediated ROS production induces apoptosis in cancer and normal cells upon stimulation, for instance by incubation with TNF-α [55], glucose [56], or anti-cancer drugs [57, 58]. On the other hand, some studies showed that cell death is not an intrinsic effect of AK2 deficiency [59], and that NOX4 could inhibit apoptosis [60, 61]. Our present results suggesting that AK2 protein and NOX4 mRNA upregulation upon irradiation could be associated with grade ≥ 2 bf+, and the finding that high RILA value (indicative of high apoptosis) is not associated with late radiotoxicity suggest a potential radiation-induced anti-apoptotic effect of these proteins. However, in order to complement the RILA assay (low PPV) and identify new toxicity biomarkers, we initially selected both patients with grade ≥ 2 bf + and < 2 bf + with low RILA score (≤16%). Therefore, the relationship between RILA and radiation-induced protein expression cannot be established based on our data. The relation between RILA values and predictive biomarkers of radiation-induced late injuries will need to be investigated to understand the link between clinical outcome and ex-vivo cellular responses to radiation.

## Conclusion

This study describes a new role of AK2 as potential lymphocyte biomarker of radiation toxicity in patients with grade ≥ 2 radiation-induced breast fibrosis. It also showed for the first time that NOX4 is the most abundant NOX isoform in PBMCs and that its expression is stimulated by irradiation, thus confirming results from previous studies [50]. We believe that our findings could provide new insight into the mechanism underlying individual radiosensitivity and the establishment of late radiation-induced toxicity, and will pave the way to additional studies.

## Additional files

Additional file 1: **Table S1.** Patients’ characteristics according to the presence or not of grade ≥ 2 radiation-induced breast fibrosis (bf+). (PDF 38 kb)

Additional file 2: **Table S2.** Primer sequences. (PDF 46 kb)

Additional file 3: **Figure S1.** Gene Ontology (GO) classification of all identified proteins (*n* = 1979). (A) Cell compartments and (B) biological processes according to the GO classification of the proteins identified by iTRAQ-nano-LC/MS/MS. (PDF 109 kb)

Additional file 4: **Figure S2.** Subcellular localization of the 1979 proteins identified by nano-LC/MS/MS analysis after iTRAQ labeling in the (A) Cytosolic, (B) Membrane, and (C) Nucleic fractions. (PDF 94 kb)

Additional file 5: **Figure S3.** Gene Ontology (GO) classification of the 23 selected proteins. (A) Cellular compartments, and (B) Biological processes according to the GO classification. (PDF 102 kb)

Additional file 6: **Table S3.** Protein selection. Protein names in bold are proteins with the highest 8Gy/0Gy ratio and chosen for confirmation by western blot analysis. P1 and P2: patients with grade ≥ 2 breast fibrosis (bf+). P3 and P4: patients without grade ≥ 2 bf + . (PDF 207 kb)

Additional file 7: **Figure S4.** qRT-PCR analysis of the AK2 mRNAs. (PDF 64 kb)

Additional file 8: **Figure S5.** Analysis of total ROS (A) and superoxide anion (change also in fig) (B) levels in PBMCs of all patients (*n* = 20; *n* = 7 with and *n* = 13 without grade ≥ 2 bf+) at 1, 6, 24, 48, 72 and 120 h post-irradiation (8 Gy). Data are the mean ± SEM; *p < 0.05 (2-tailed Mann-Whitney test). (PDF 70 kb)

Additional file 9: **Figure S6.** Measurement of intracellular total ROS (A) and superoxide anion (B) in patients with grade < 2 bf + (*n* = 13, circles) and grade ≥ 2 bf + (*n* = 7, squares) at 1, 6, 24, 48, 72 and 120 h post-irradiation (8 Gy). Data are the mean ± SEM; *p < 0.05 (2-tailed Mann-Whitney test). (PDF 91 kb)

Additional file 10: **Figure S7.** mRNA expression by qRT-PCR analysis of NOX family members in all patients (*n* = 20; *n* = 7 with and *n* = 13 without grade ≥ 2 bf+) at 24 h after irradiation (8 Gy). ND not detected (below threshold). (PDF 883 kb)

## Data Availability

The datasets used and/or analyzed during the current study are available from the corresponding author on reasonable request.

## Abbreviations

AK2: Adenylate Kinase 2
bf+: Breast fibrosis
FDR: False Discovery Rate
GO: Gene Ontology
i-TRAQ: isobaric Tags for Relative and Absolute Quantitation
NOXs: NADPH oxidases
PBMCs: Peripheral Blood Mononuclear Cells
RILA: Radiation-Induced Lymphocyte Apoptosis
ROS: Reactive Oxygen Species
RT: Radiation Therapy
