# Supplementary material for: Quantitative proteomic analysis reveals AK2 as potential biomarker for late normal tissue radiotoxicity
Source: Radiat Oncol. 2019 Aug 9;14:142. doi: 10.1186/s13014-019-1351-8 (PMC6688300; doi:10.1186/s13014-019-1351-8)
Supplement: Supplementary file 1 — Table S1. Patients’ characteristics according to the presence or not of grade ≥ 2 radiation-induced breast fibrosis (bf+). (PDF 38 kb) [file 13014_2019_1351_MOESM1_ESM.pdf]

Supplementary Table S1. Patients' characteristics according to radiation-induced breast fibrosis (bf+)

		Grade <2 RISF (n=23)		Grade ≥2 RISF (n=7)	
**Age (years)**		61	(53–80)	59	(51– 65)
**WHO**	0	23	(100.0%)	7	(100.0%)
**Weight (kg)***		69.0	(48– 97)	66	(55– 80)
**Height (cm)***		160	(149 – 170)	156	(155 – 160)
**Body Mass Index, BMI (kg/m²)***		26.0	(18.6 – 34.9)	26.2	(25.8 – 31.6)
**BMI**	low<br>normal<br>overweight<br>obese	0<br>8<br>8<br>5	(0.0%)<br>(38.1%)<br>(38.1%)<br>(23.8%)	0<br>0<br>2<br>1	(0.0%)<br>(0.0%)<br>(66.7%)<br>(33.3%)
**Tumor location**	Left<br>Right	9<br>14	(39.1%)<br>(60.9%)	6<br>1	(85.7%)<br>(14.3%)
**Breast volume**	Small volume<br>Large volume	7<br>15	(31.8%)<br>(68.2%)	1<br>5	(16.7%)<br>(83.3%)
**Smoker**	No<br>Ex-smoker<br>Smoker	17<br>0<br>6	(73.9%)<br>(0.0%)<br>(26.1%)	6<br>0<br>1	(85.7%)<br>(0.0%)<br>(14.3%)
**Tumor (T)**	0<br>1<br>2	7<br>12<br>3	(31.8%)<br>(54.5%)<br>(13.6%)	2<br>4<br>1	(28.6%)<br>(57.1%)<br>(14.3%)
**Node status**	0<br>1<br>2	20<br>3<br>0	(87.0%)<br>(13.0%)<br>(0.0%)	5<br>2<br>0	(71.4%)<br>(28.6%)<br>(0.0%)
**Resected tumor volume (mm³)***		21	235.95 (9.9 – 9929.0)	3	42.6 (6.7 – 756.0)
**Diabetes mellitus/ Hypertension**	No<br>Yes	19<br>4	(82.6%)<br>(17.4%)	6<br>1	(85.7%)<br>(14.3%)
**RILA** (baseline)**	≤16%<br>16-24%<br>>24%	13<br>6<br>4	(56.5%)<br>(26.1%)<br>(17.4%)	6<br>1<br>0	(85.7%)<br>(14.3%)<br>(0.0%)
**Radiation Boost**	No<br>Yes	9<br>14	(39.1%)<br>(60.9%)	0<br>7	(0.0%)<br>(100.0%)
**Adjuvant chemotherapy**	No<br>Yes	18<br>5	(78.3%)<br>(21.7%)	3<br>4	(42.9%)<br>(57.1%)

Abbreviations: *median (range); **RILA, Radiation-induced lymphocyte apoptosis
