# Supplementary material for: Quantitative proteomic analysis reveals AK2 as potential biomarker for late normal tissue radiotoxicity
Source: Radiat Oncol. 2019 Aug 9;14:142. doi: 10.1186/s13014-019-1351-8 (PMC6688300; doi:10.1186/s13014-019-1351-8)
Supplement: Supplementary file 2 — Table S2. Primer sequences. (PDF 46 kb) [file 13014_2019_1351_MOESM2_ESM.pdf]

Supplementary Table S2. **List of primer sequences**

Gene	Forward	Reverse
AK2	CTACTGGGGACATGCTGAGG	TTTTGCACAAGGGGGTCTCC
ANXA1	GGGCCTTGGAACTGATGAAGA	CAGATCGGTCACCCTTAGCA
APEX1	GCATAGGCGATGAGGAGCAT	GAGGTCTCCACACAGCACAA
HSPA8	CGGGCTTGTGATTGGGTCTT	GCCACCCTGCCTCTTATACC
IDH2	CTCAAGTCTTCGGGTGGCTT	GCCTCAGCCTCAATCGTCTT
NOX1	TCTTATGTGGCCCTCGGACT	TGCTCAAACCTGACGAGACC
NOX2	GGGAACTGGGCTGTGAATGA	CCAGTGCTGACCCAAGAAGT
NOX3	ACCGGCTGGGATGAAAATCA	CAATACTGCTGCTGGGGTGA
NOX4	AGTGAGGAGCTGAACTTGCTC	TATTTGCCTGGAGTGCTTGC
NOX5	GGTGGGTGACTCAGCAGTTT	GGCAAAGAATCGCTCTGCAA
DUOX1	GAGAAACCGCACAGTGTTGG	CGGGATGCGAATGTTGAGGA
DUOX2	GCTGCCTTCCCTTAGTGAGTC	ATCGCTGGCACTCCATCTTT
β2m	TGCTGTCTCCATGTTTGATGTATCT	TCTCTGCTCCCCACCTCTAAGT
RS9	CGGCCCGGGAGCTGTTGACG	CTGCTTGCGGACCCTAATGTGACG
