# Supplementary figures and images for: Quantitative proteomic analysis reveals AK2 as potential biomarker for late normal tissue radiotoxicity
Source: Radiat Oncol. 2019 Aug 9;14:142. doi: 10.1186/s13014-019-1351-8 (PMC6688300; doi:10.1186/s13014-019-1351-8)

Figure S1

A

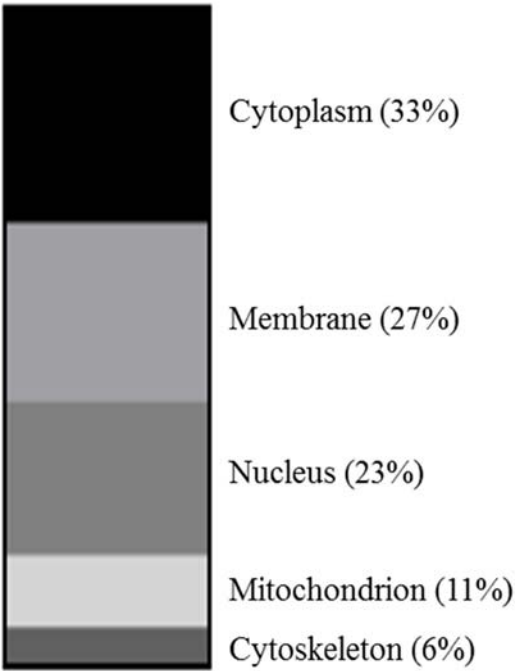

B

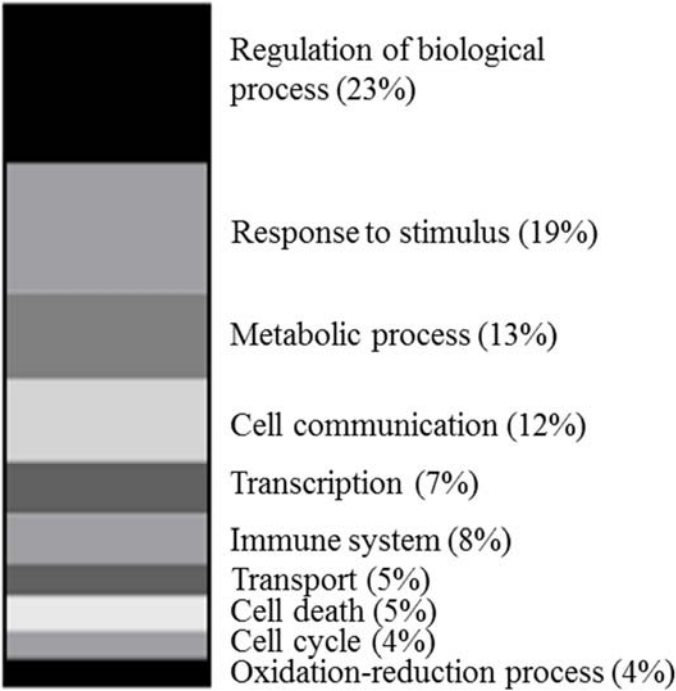

Supplement: Supplementary file 3 — Figure S1. Gene Ontology (GO) classification of all identified proteins (n = 1979). (A) Cell compartments and (B) biological processes according to the GO classification of the proteins identified by iTRAQ-nano-LC/MS/MS. (PDF 109 kb) [file 13014_2019_1351_MOESM3_ESM.pdf]

Figure S2

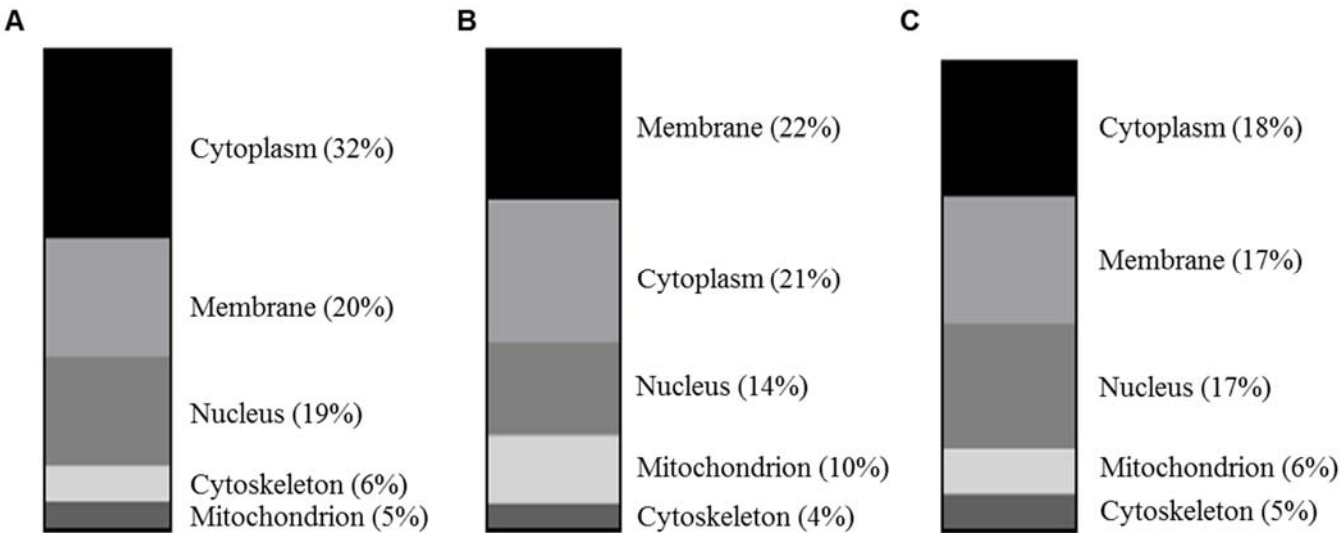

Supplement: Supplementary file 4 — Figure S2. Subcellular localization of the 1979 proteins identified by nano-LC/MS/MS analysis after iTRAQ labeling in the (A) Cytosolic, (B) Membrane, and (C) Nucleic fractions. (PDF 94 kb) [file 13014_2019_1351_MOESM4_ESM.pdf]

Figure S3

A

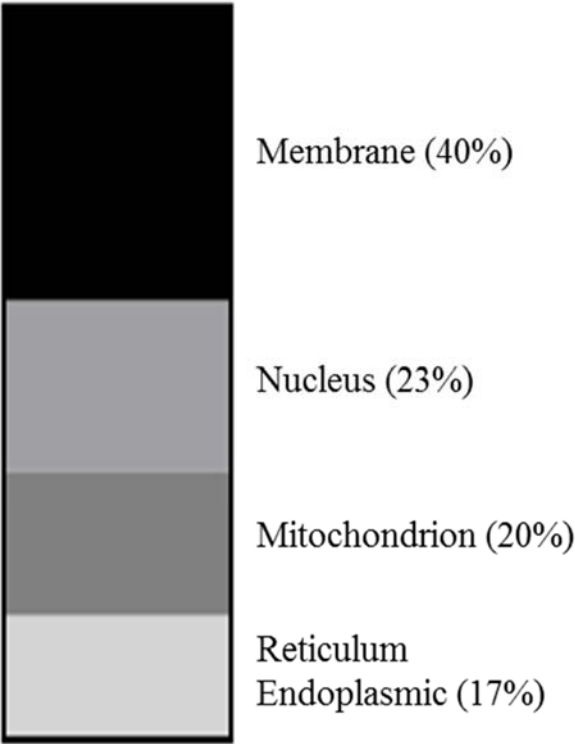

B

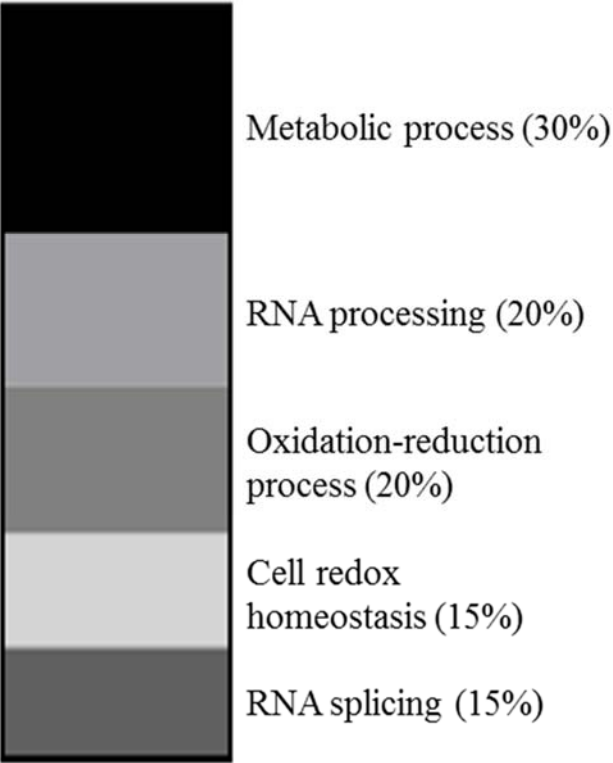

Supplement: Supplementary file 5 — Figure S3. Gene Ontology (GO) classification of the 23 selected proteins. (A) Cellular compartments, and (B) Biological processes according to the GO classification. (PDF 102 kb) [file 13014_2019_1351_MOESM5_ESM.pdf]

Figure S4

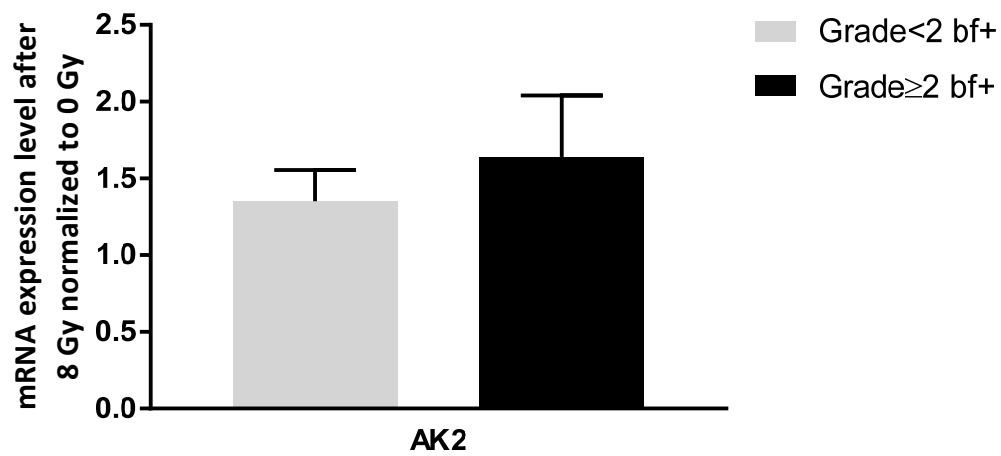

Supplement: Supplementary file 7 — Figure S4. qRT-PCR analysis of the AK2 mRNAs. (PDF 64 kb) [file 13014_2019_1351_MOESM7_ESM.pdf]

Figure S5

A

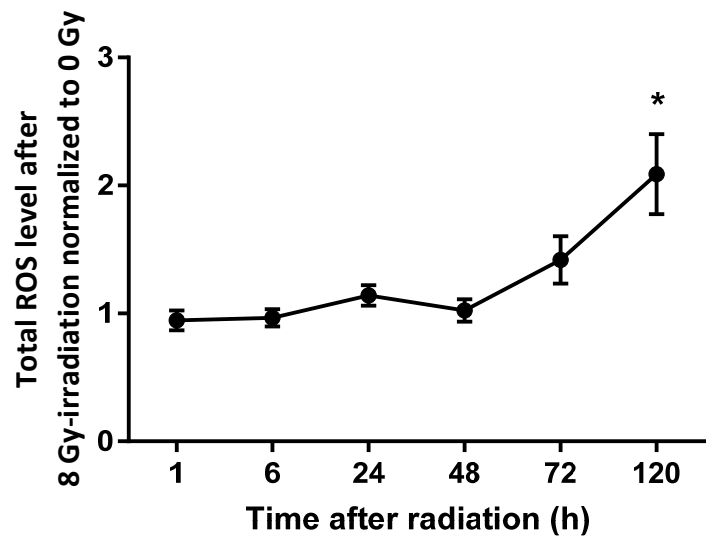

B

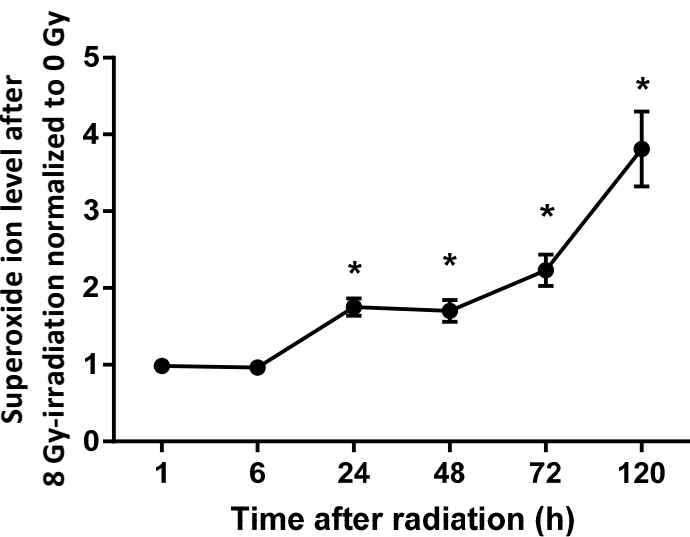

Supplement: Supplementary file 8 — Figure S5. Analysis of total ROS (A) and superoxide anion (change also in fig) (B) levels in PBMCs of all patients (n = 20; n = 7 with and n = 13 without grade ≥ 2 bf+) at 1, 6, 24, 48, 72 and 120 h post-irradiation (8 Gy). Data are the mean ± SEM; *p < 0.05 (2-tailed Mann-Whitney test). (PDF 70 kb) [file 13014_2019_1351_MOESM8_ESM.pdf]

Figure S6

**A**

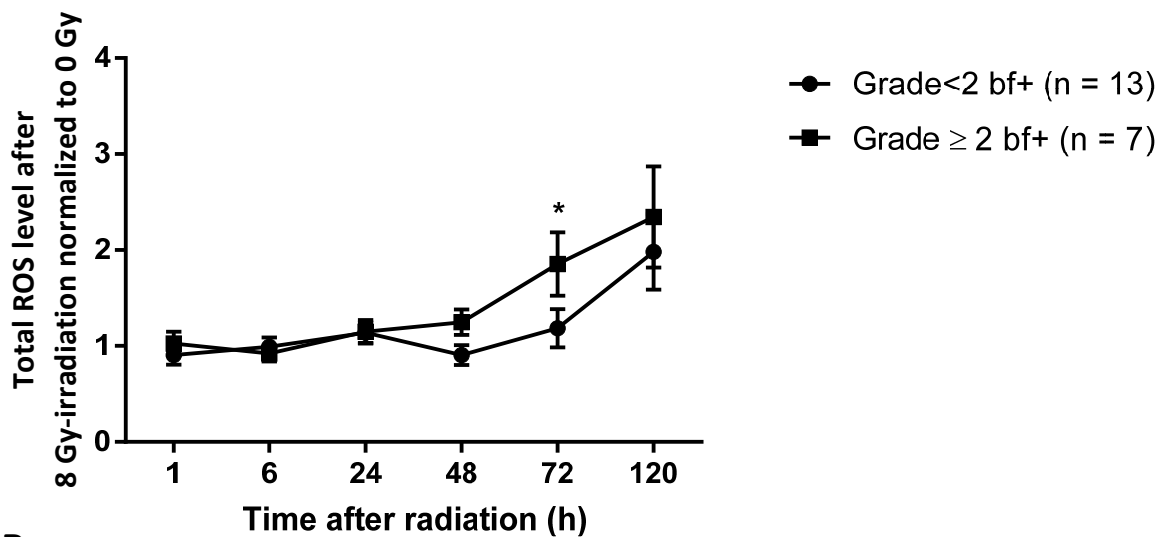

**B**

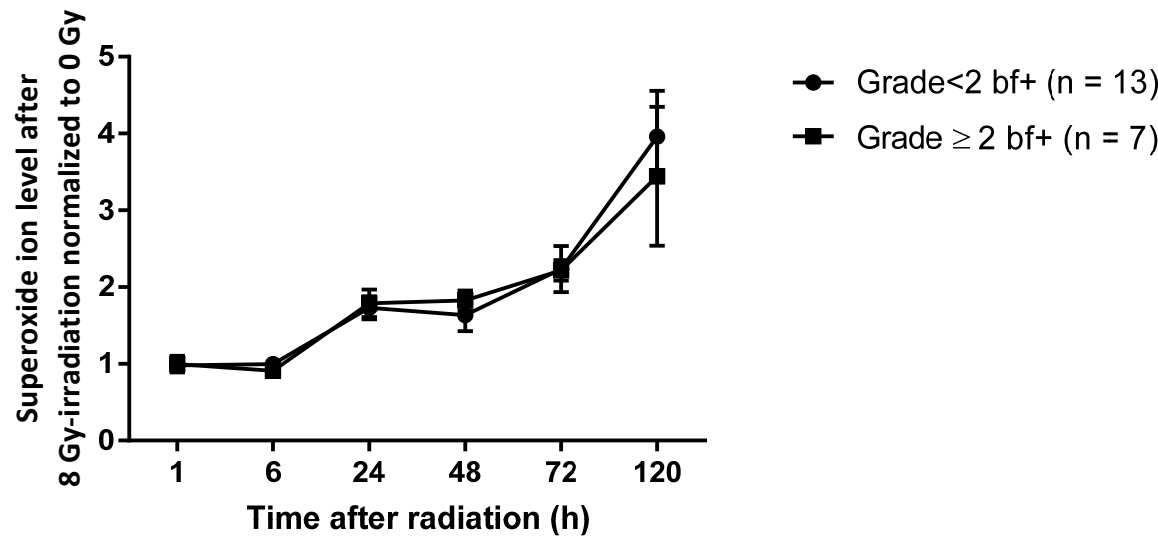

Supplement: Supplementary file 9 — Figure S6. Measurement of intracellular total ROS (A) and superoxide anion (B) in patients with grade < 2 bf + (n = 13, circles) and grade ≥ 2 bf + (n = 7, squares) at 1, 6, 24, 48, 72 and 120 h post-irradiation (8 Gy). Data are the mean ± SEM; *p < 0.05 (2-tailed Mann-Whitney test). (PDF 91 kb) [file 13014_2019_1351_MOESM9_ESM.pdf]

Figure S7

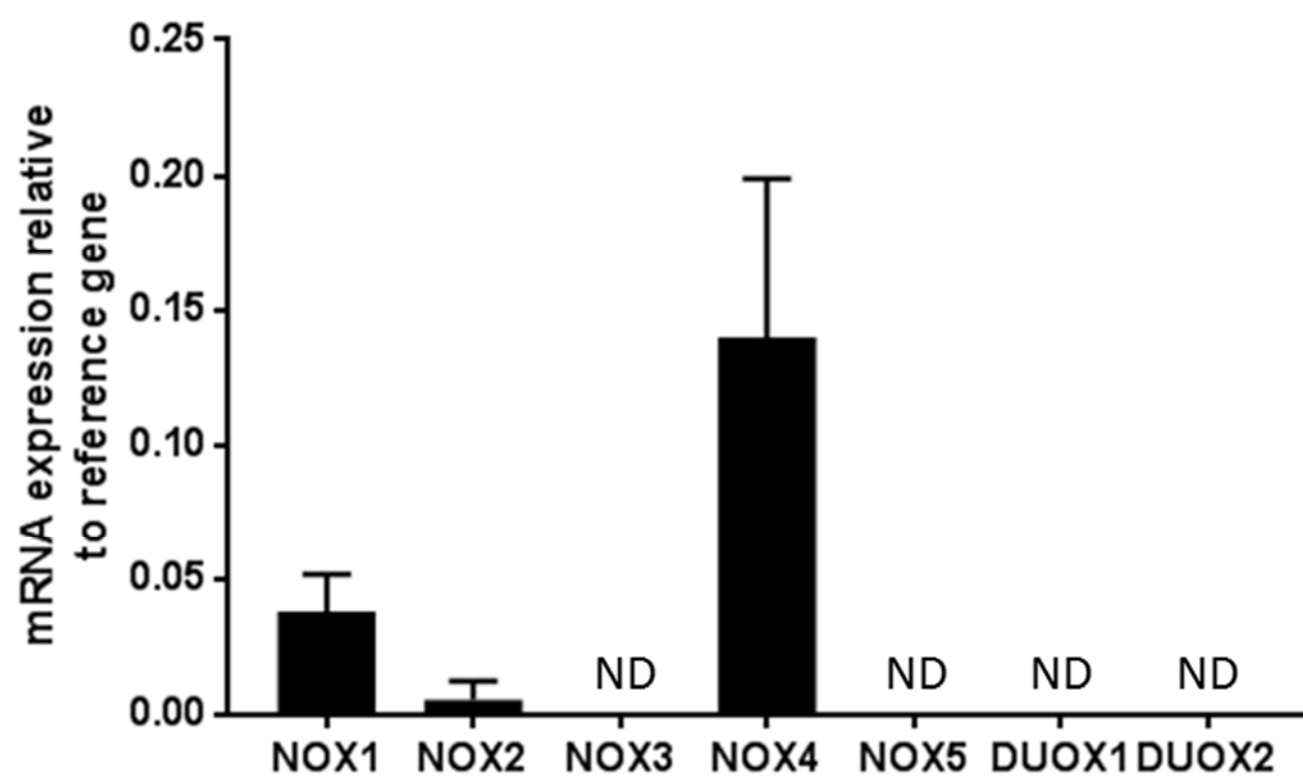

Supplement: Supplementary file 10 — Figure S7. mRNA expression by qRT-PCR analysis of NOX family members in all patients (n = 20; n = 7 with and n = 13 without grade ≥ 2 bf+) at 24 h after irradiation (8 Gy). ND not detected (below threshold). (PDF 883 kb) [file 13014_2019_1351_MOESM10_ESM.pdf]
