# Supplementary material for: Quantitative proteomic analysis reveals AK2 as potential biomarker for late normal tissue radiotoxicity
Source: Radiat Oncol. 2019 Aug 9;14:142. doi: 10.1186/s13014-019-1351-8 (PMC6688300; doi:10.1186/s13014-019-1351-8)
Supplement: Supplementary file 6 — Table S3. Protein selection. Protein names in bold are proteins with the highest 8Gy/0Gy ratio and chosen for confirmation by western blot analysis. P1 and P2: patients with grade ≥ 2 breast fibrosis (bf+). P3 and P4: patients without grade ≥ 2 bf + . (PDF 207 kb) [file 13014_2019_1351_MOESM6_ESM.pdf]

Supplementary Table S3: Protein selection. Protein names in bold are proteins chosen for further western-blot validation. P1 and P2: patients with grade ≥2 breast fibrosis (bf+). P3 and P4: patients without grade ≥2 bf+.

Uniprot accession	Gene symbol	Protein name	8 Gy				0 Gy				8Gy/0Gy			
			P1/P3	P1/P4	P2/P3	P2/P4	P1/P3	P1/P4	P2/P3	P2/P4	P1/P3	P1/P4	P2/P3	P2/P4
Q09666	AHNAK	Neuroblast differentiation-associated protein AHNAK	7.18	1.91	1.96	0.46	1.16	1.27	0.54	0.61	6.2	1.5	3.6	0.8
P35579	MYH9	Myosin-9	0.3	0.28	0.63	0.57	0.76	0.34	1.13	0.52	0.4	0.8	0.6	1.1
P08133	ANXA6	Annexin A6	0.92	0.64	0.47	0.34	0.3	0.2	0.7	0.45	3	3.2	0.7	0.8
P22626	HNRNPA2B1	Heterogeneous nuclear ribonucleoproteins A2/B1	0.41	0.56	0.3	0.43	0.97	0.56	1.1	0.65	0.4	1	0.3	0.7
P10412	HIST1H1E	Histone H1.4	0.48	0.59	0.77	0.96	0.9	0.49	0.95	0.54	0.5	1.2	0.8	1.8
P30101	PDIA3	Protein disulfide-isomerase A3	0.7	0.73	0.48	0.51	0.89	0.53	0.73	0.49	0.8	1.4	0.7	1
P11142	HSPA8	**Heat shock cognate 71 kDa protein**	0.9	0.96	1.75	1.79	0.36	0.32	0.99	0.96	**2.5**	**3**	**1.8**	**1.9**
P04083	ANXA1	**Annexin A1**	4.53	1.6	1.39	0.47	2.23	0.64	0.87	0.25	**2**	**2.5**	**1.6**	**1.9**
P40939	HADHA	Trifunctional enzyme subunit alpha, mitochondrial	1.61	1.69	0.63	0.66	2.07	2.01	1.71	1.77	0.8	0.8	0.4	0.4
P27695	APEX1	**DNA-(apurinic or apyrimidinic site) lyase**	1.94	1.64	1.24	1.01	1.34	1.09	1.46	1.16	1.4	1.5	0.8	0.9
Q9BSJ8	ESYT1	Extended synaptotagmin-1	2.15	1.79	1.06	1	6.43	1.89	2.15	0.7	0.3	0.9	0.5	1.4
P04899	GNAI2	Guanine nucleotide-binding protein G(i) subunit alpha-2	1.96	2.81	0.33	0.47	11.8	0.97	11.48	0.76	0.2	2.9	0	0.6
P49411	TUFM	Elongation factor Tu, mitochondrial	1.67	2.36	1.02	1.34	1.8	1.28	1.22	0.9	0.9	1.8	0.8	1.5
Q1KMD3	HNRNPUL2	Heterogeneous nuclear ribonucleoprotein U-like protein 2	1.92	3.02	0.9	1.51	1.42	1.56	1.07	1.14	1.4	1.9	0.8	1.3
P54819	AK2	**Adenylate kinase 2, mitochondrial**	2.23	4.7	1.5	3.63	1.08	0.79	1.19	0.79	**2.1**	**6**	**1.3**	**4.6**
P02545	LMNA	Prelamin-A/C	4.41	2.01	2.86	1.24	1.67	3.19	1.37	2.58	2.6	0.6	2.1	0.5
P48735	IDH2	**Isocitrate dehydrogenase [NADP], mitochondrial**	2.49	2.15	1.51	1.43	0.81	0.66	0.74	0.59	**3.1**	**3.3**	**2.1**	**2.4**
P21912	SDHB	Succinate dehydrogenase [ubiquinone] iron-sulfur subunit, mitochondrial	1.29	0.98	0.6	0.47	1.01	1.12	0.82	0.97	1.3	0.9	0.7	0.5
P13804	ETFA	Electron transfer flavoprotein subunit alpha, mitochondrial	4.02	2.63	1.85	1.33	0.81	1.66	0.84	1.5	5	1.6	2.2	0.9
Q6P2Q9	PRPF8	Pre-mRNA-processing-splicing factor 8	1.64	1.69	0.59	0.61	1.25	1.28	0.92	0.93	1.3	1.3	0.6	0.7
P09382	LGALS1	**Galectin-1**	6.98	3.63	4.61	2.42	1.14	0.58	1.12	0.57	**6.1**	**6.3**	**4.1**	**4.2**
P30481	HLA-B	HLA class I histocompatibility antigen, B-44 alpha chain	0.17	0.11	0.32	0.17	0.89	0.31	0.69	0.25	0.2	0.3	0.5	0.7
Q08170	SRSF4	Serine/arginine-rich splicing factor 4	1.63	0.99	0.19	0.11	0.52	0.96	0.19	0.81	3.1	1	1	0.1

Ratio: >3 >2 >1.5 <0.66 <0.5 <0.33
